# Enhancing Development Through Teleoccupational Therapy in Children With Cortical Visual Impairment: A Quasiexperimental Study

**DOI:** 10.1155/oti/3415529

**Published:** 2026-02-22

**Authors:** Safa Heybet, Seval Cevher Eylen

**Affiliations:** ^1^ Department of Therapy and Rehabilitation, Vocational School, Biruni University, Istanbul, Turkey, biruni.edu.tr; ^2^ Department of Occupational Therapy, Institute of Graduate Studies, Biruni University, Istanbul, Turkey, biruni.edu.tr

**Keywords:** cortical visual impairment, developmental skills, pediatric rehabilitation, play skills, teleoccupational therapy

## Abstract

**Purpose:**

This study was aimed at investigating the effectiveness of a teleoccupational therapy intervention program in improving developmental and play skills in children diagnosed with cortical visual impairment (CVI). The intervention was structured based on the person–environment–occupation (PEO) model.

**Materials and Methods:**

A quasiexperimental, pretest–posttest study design was employed with eight children aged 4–6 diagnosed with CVI. The intervention consisted of 12 weekly online occupational therapy sessions, focused on visual perception, motor skills, and environmental adaptation strategies. Outcome measures included the Ankara Developmental Screening Inventory (ADSI) and the Revised Knox Preschool Play Scale (RKPPS). Data were analyzed using the Wilcoxon signed‐rank test.

**Results:**

Significant improvements were observed in motor accuracy, visual attention, and sensory processing after the intervention (*p* < 0.05). Parental reports indicated increased engagement in purposeful play and daily routines. No adverse effects were reported.

**Conclusion:**

The findings support the feasibility and effectiveness of teleoccupational therapy based on the PEO model in enhancing developmental and play skills in children with CVI. The approach offers a viable alternative when in‐person services are limited.

**Implications for Rehabilitation**

• Teleoccupational therapy can be an effective and accessible intervention for children with cortical visual impairment (CVI) when in‐person services are limited.
• Developmental and play‐based interventions delivered online improve engagement in daily routines and motor planning skills.
• Environmental adaptation strategies guided through telehealth empower caregivers to support therapy goals at home.
• The person–environment–occupation (PEO) model provides a valuable framework for tailoring remote rehabilitation interventions in pediatric populations.

## 1. Background

Cerebral visual impairment, also referred to as CVI in some contexts, is a neurodevelopmental visual disorder resulting from damage to the postgeniculate visual pathways and associated visual processing networks. Contemporary definitions acknowledge that CVI may coexist with mild abnormalities of the anterior visual pathways, and normal ocular structure is no longer considered a prerequisite for diagnosis [1, 2]. Cortikal visual impairment (CVI)is frequently associated with neurological conditions such as cerebral palsy, epilepsy, hypoxic–ischemic encephalopathy, and structural brain abnormalities, which may significantly affect visual processing and functional development [3]. Unlike ocular‐based visual impairments, CVI requires a different therapeutic approach that integrates environmental modifications and structured interventions to enhance functional vision and overall development [4].

Play is considered an essential primary occupation of childhood and represents a fundamental context through which children develop cognitive, social, and motor skills [5]. Children with visual impairment often exhibit delays in play behavior, particularly in symbolic play, object manipulation, and social interactions [6]. These challenges may lead to secondary impairments in communication and independence, emphasizing the need for structured occupational therapy interventions tailored to the unique needs of children with CVI [7].

Telerehabilitation has gained increasing attention as a means of delivering therapeutic interventions remotely, overcoming accessibility barriers, and providing individualized therapy in a home environment [8]. Studies indicate that remote interventions can be effective in supporting children with various disabilities, including those with visual impairments [9]. The PEO model provides a valuable framework for occupational therapy by emphasizing the interaction between the individual, their surroundings, and their daily activities [10].

This study is aimed at exploring the impact of online occupational therapy interventions, grounded in the PEO model, on the developmental and play skills of children with CVI. By addressing the unique challenges posed by CVI through a structured, telerehabilitation‐based approach, this research seeks to contribute to the growing body of evidence supporting remote occupational therapy interventions for pediatric populations with visual impairments.

## 2. Methods

### 2.1. Study Design

This study employed a quasiexperimental pretest–posttest design to examine the effectiveness of online occupational therapy interventions on the developmental and play skills of children diagnosed with CVI. The intervention was conducted within the framework of the PEO model to ensure a holistic approach to therapy.

### 2.2. Participants

A total of 8 children (4 males, 4 females) aged 48–72 months with a confirmed diagnosis of CVI were included in this study. The diagnosis of CVI was confirmed based on existing clinical records from pediatric ophthalmology and child neurology services. Diagnostic information was obtained from medical reports provided by the families at enrollment. No additional neuropsychological testing was conducted as part of the study. The absence of severe cognitive impairment was determined based on clinical records, parental reports, and functional developmental performance observed during the Ankara Developmental Screening Inventory (ADSI) assessment. Participants were recruited through occupational therapy clinics and online support groups for children with visual impairments. This study employed a single‐group quasiexperimental pretest–posttest design. All participants received the teleoccupational therapy intervention, and no control group was included. The inclusion and exclusion criteria were as follows.

Inclusion criteria include the following:

• Diagnosis of CVI confirmed by a pediatric ophthalmologist and neurologist.
• Ability to participate in online therapy sessions with parental support.
• No additional severe physical or cognitive impairments.

Exclusion criteria include the following:

• Presence of progressive neurological disorders.
• Severe behavioral issues preventing participation in online sessions.
• Previous structured play‐based therapy within the last 6 months.

All participants′ parents provided informed consent before enrollment. This study received ethical approval from the Institutional Review Board of Biruni University (Approval No. 2015‐KAEK‐61‐22‐08, 09.03.2022).

### 2.3. Intervention

Participants underwent an 8‐week telerehabilitation‐based occupational therapy program, with one session per week lasting approximately 45 min. The teleoccupational therapy intervention was individually tailored and structured into three core components: (1) environmental modifications, (2) play‐based occupational therapy activities, and (3) parental training and support.

### 2.4. Environmental Modifications

Several adjustments were implemented in the home environment to maximize the children′s visual processing abilities. Room lighting was optimized to avoid dim or overly bright conditions, and uniform light sources that did not cast shadows on the child′s face were preferred. To enhance visual attention and object discrimination, toys and learning materials were presented against high‐contrast backgrounds. The placement of objects was organized to facilitate visual accessibility; frequently used items were positioned at eye level and within easy reach of the child. In addition, distracting elements were minimized by removing unnecessary objects and maintaining a simplified arrangement that reduced visual clutter. These environmental adaptations were designed to be sustainable for families at home, and therapists provided feedback after each session through photographs or video recordings submitted by the parents.

### 2.5. Play‐Based Occupational Therapy Activities

During telerehabilitation sessions, play‐based activities were designed according to the children′s developmental level and age. The program included sensory play activities (e.g., exploration with textured objects, tactile stimulation), fine motor tasks (e.g., grasping objects, placing small items, and block construction), and gross motor activities (e.g., reaching, bilateral play, and directional movement). In addition, cognitive and social play components were incorporated, such as storytelling and guided symbolic play, which promoted imagination, social interaction, and visual–motor integration. Each activity was tailored to the child′s individual capacity, with tasks gradually increasing in complexity. The therapist guided the activities in real time via Zoom and provided immediate suggestions to parents when necessary to ensure active participation and engagement of the child in the play tasks.

### 2.6. Parental Training and Support

Parental involvement was considered a fundamental component of the intervention. At the end of each session, parents received practical strategies to facilitate their children′s play and daily activities in ways that supported visual perception. Simple, home‐based play suggestions were shared (e.g., matching tasks with high‐contrast materials, imitation games, and tactile exploration activities). Parents were also guided on how to encourage independent play, strengthen social interactions, and maintain the child′s motivation during tasks. These educational components were delivered through weekly online meetings, where parents′ questions were addressed, and supplementary written and visual materials were provided to support implementation at home. To ensure fidelity, sessions were video‐recorded, reviewed by the therapist, and constructive feedback was regularly provided to the parents.

### 2.7. Outcome Measures

Developmental and play‐related outcomes were assessed using two standardized instruments: the ADSI and the Revised Knox Preschool Play Scale (RKPPS). Both assessments were administered by the same independent occupational therapist at baseline (preintervention) and after completion of the intervention (postintervention).

### 2.8. ADSI

The ADSI was administered to evaluate the developmental performance of the participating children across multiple domains. The ADSI is a standardized and widely used developmental assessment tool designed for children between 0 and 6 years of age. It provides a structured evaluation of four major developmental domains: language–cognitive skills, fine motor skills, gross motor skills, and social–self‐care abilities. The instrument includes age‐specific items that assess a child′s capacity to perform daily functional activities, problem‐solving tasks, and interactive behaviors.

The ADSI was administered by the same independent occupational therapist both before and after the intervention.

In this study, the ADSI was administered individually to each child by an independent occupational therapist who was blinded to the intervention process. Parents were involved in the administration, as the inventory requires caregiver input to ensure accurate reflection of the child′s behaviors and skills in natural contexts. The assessment took approximately 25–30 min per child and was conducted via structured online sessions, supported by parental observations and reports.

Scoring was carried out according to the standardized ADSI manual. Each item was coded as either achieved or not achieved, and the cumulative scores were calculated for each developmental domain. Higher scores indicated more advanced developmental performance. Both preintervention and postintervention assessments were completed to capture changes over the 8‐week therapy program. In order to ensure reliability, the occupational therapist reviewed the responses with parents after the sessions to clarify any ambiguous observations.

The ADSI was selected as a primary outcome measure because of its strong validity and reliability in Turkish pediatric populations and its sensitivity to developmental changes in children with neurological and sensory impairments, including visual impairments. Its comprehensive scope allowed for the detection of subtle improvements in developmental domains that were directly targeted by the intervention, such as fine motor dexterity, gross motor coordination, and language–cognitive functioning.

### 2.9. RKPPS

The RKPPS measures play behaviors across four dimensions: space management, material management, pretense‐symbolic play, and participation. It provides a qualitative and quantitative analysis of play skills. Both assessments were administered pre‐ and postintervention by an independent occupational therapist blinded to the intervention process.

### 2.10. Data Analysis

Statistical analysis (median, 25th and 75th percentiles) was conducted using SPSS (v.26). Due to the small sample size, a nonparametric Wilcoxon signed‐rank test was used to compare pre‐ and postintervention scores for both ADSI and RKPPS. A significance level of *p* < 0.05 was considered statistically significant.

## 3. Results

A total of 8 children (4 males, 4 females) diagnosed with CVI participated in this study. The median age of the participants was 51.00 months. All children had confirmed CVI diagnoses and exhibited varying degrees of visual processing difficulties. No participants had severe motor or cognitive impairments that would prevent engagement in occupational therapy interventions. The demographic and clinical characteristics of the participants, including age, gender, and visual processing difficulties, are presented in Table 1.

**Table 1:** Demographic and clinical characteristics of participants.

**Parameter**	**Median**	**25th percentile**	**75th percentile**
Age (month)	51.00	48.50	66.00
**Parameter**	**Subparameter**	**Number**	**Percent**
Use of refractive glasses	Use	6	75%
	Do not use	2	25%
Type of school	Preschool or nursery	2	25%
	Special education and rehabilitation center	6	75%
Number of siblings	No sibling	2	25%
	1	4	50%
	2	1	12.5%
	3+	1	12.5%

*Note:* Values are presented as median (25th–75th percentile).

Following the 8‐week telerehabilitation‐based intervention, significant improvements were observed in developmental and play skills. Pre‐ and postintervention comparisons using the Wilcoxon signed‐rank test revealed statistically significant enhancements in multiple domains of the ADSI and the RKPPS. A summary of the pre‐ and postintervention scores for the ADSI and RKPPS, along with statistical significance values, is shown in Table 2.

**Table 2:** Pretest and posttest scores for developmental and play skills.

Assessment	Subparameter	Pretest median	25th–75th	Posttest median	25th–75th	*Z*	*p* value
Ankara Developmental Screening Inventory (AGTE)	Cognitive	26.50	15.00–42.75	33.50	20.00–49.00	−2.375	**0.018**
	Fine motor	11.00	9.25–15.75	13.00	12.00–20.00	−2.388	**0.017**
	Gross motor	11.00	9.00–23.00	13.00	10.50–24.00	−2.640	**0.008**
	Social	28.00	16.00–30.00	28.50	16.00–31.50	−1.633	0.102
	Total	76.50	49.25–111.50	87.00	57.00–124.50	−2.527	**0.012**
Knox Preschool Play Scale (RKPPS)	Space management	50.00	48.00–54.50	51.00	48.00–55.00	−1.414	1.000
	Gross motor	48.00	46.00–55.50	50.00	48.00–56.50	−1.414	1.000
	Interest	51.00	48.50–56.50	51.00	48.50–56.50	0.000	1.000
	Material management	48.00	44.50–51.00	56.00	50.50–56.00	−2.549	**0.011**
	Manipulation	48.00	48.00–55.50	50.00	50.00–56.00	−2.449	**0.014**
	Construction	48.00	46.50–55.50	50.00	47.00–56.00	−2.000	**0.046**
	Purpose	50.00	48.00–56.00	50.00	48.50–56.00	−1.000	0.317
	Attention	46.00	46.00–55.00	50.00	50.00–59.00	−2.828	**0.005**
	Pretense/symbolic	48.00	46.50–54.50	52.00	48.00–55.00	−1.633	0.102
	Imitation	48.00	48.00–55.50	50.00	48.00–56.00	−1.732	0.083
	Dramatization	53.00	48.00–57.50	50.00	48.00–56.00	−1.414	0.157
	Participation	51.00	48.00–58.00	51.00	48.00–58.00	0.000	1.000
	Type	49.00	46.50–56.00	49.00	46.50–56.00	0.000	1.000
	Cooperation	49.00	48.00–56.00	49.00	48.00–56.00	−1.500	0.089
	Humor	50.00	48.00–56.00	50.00	48.00–56.00	−1.500	0.089
	Language	50.00	48.00–56.00	50.00	48.00–56.00	−0.750	0.091

*Note:* Wilcoxon signed‐rank test, *p* < 0.05. Values presented in bold indicate statistically significant results (*p* < 0.05).

In terms of developmental outcomes, cognitive development scores showed a significant increase (*Z* = −2.375, *p* = 0.018), suggesting improved verbal expression and cognitive processing abilities. Fine motor skill scores also increased significantly (*Z* = −2.388, *p* = 0.017), indicating enhanced dexterity and object manipulation. Similarly, gross motor skills improved after the intervention (*Z* = −2.640, *p* = 0.008), reflecting better coordination and movement control. The correlation coefficients between developmental domains and play skills improvements are displayed in Table 3, highlighting the strong relationships between cognitive, motor, and play‐based outcomes.

**Table 3:** Correlations between changes in developmental domains (ADSI) and play skills (RKPPS).

Parameter		AGTE				
RKPPS		Cognitive	Fine motor	Gross motor	Social	Total
Space management	*r*	0.087	0.494	−0.268	0.191	0.086
	*p*	0.838	0.214	0.522	0.650	0.839
Gross motor	*r*	−0.062	0.367	−0.395	0.013	−0.062
	*p*	0.884	0.371	0.333	0.976	0.885
Interest	*r*	0.135	0.475	−0.252	0.277	0.134
	*p*	0.750	0.234	0.548	0.507	0.751
Material management	*r*	0.349	0.658	−0.020	0.431	0.347
	*p*	0.397	0.076	0.963	0.287	0.400
Manipulation	*r*	−0.458	−0.096	**−0.776** ^∗^	−0.356	−0.455
	*p*	0.254	0.820	**0.023**	0.387	0.258
Construction	*r*	0.039	0.342	−0.318	0.186	0.039
	*p*	0.927	0.406	0.443	0.660	0.928
Purpose	*r*	0.039	0.342	−0.318	0.186	0.039
	*p*	0.927	0.406	0.443	0.660	0.928
Attention	*r*	−0.510	−0.130	**−0.784** ^∗^	−0.392	−0.507
	*p*	0.197	0.759	**0.021**	0.337	0.200
Pretense/symbolic	*r*	0.051	0.465	−0.273	0.143	0.050
	*p*	0.905	0.246	0.513	0.736	0.906
Imitation	*r*	0.051	0.465	−0.273	0.143	0.050
	*p*	0.905	0.246	0.513	0.736	0.906
Dramatization	*r*	0.051	0.465	−0.273	0.143	0.050
	*p*	0.905	0.246	0.513	0.736	0.906
Participation	*r*	0.341	0.683	−0.025	0.375	0.339
	*p*	0.408	0.062	0.953	0.360	0.411
Type	*r*	−0.198	0.201	−0.506	−0.101	−0.196
	*p*	0.639	0.633	0.200	0.811	0.641
Cooperation	*r*	−0.013	0.369	−0.398	0.106	−0.013
	*p*	0.976	0.369	0.329	0.803	0.976
Humor	*r*	0.051	0.465	−0.273	0.143	0.050
	*p*	0.905	0.246	0.513	0.736	0.906
Language	*r*	0.051	0.465	−0.273	0.143	0.050
	*p*	0.905	0.246	0.513	0.736	0.906

*Note:* Spearman correlation test. Values presented in bold indicate statistically significant correlations between variables (*p* < 0.05).

^∗^Statistically significant correlations (*p* < 0.05).

Regarding play skill development, material management scores showed a statistically significant improvement (*Z* = −2.549, *p* = 0.011), suggesting that children demonstrated better handling and interaction with objects during play. Also, manipulation (*Z* = −2.449, *p* = 0.014), construction (*Z* = −2.000, *p* = 0.046), and attention (*Z* = −2.828, *p* = 0.005) had statistically significant results.

Spearman′s rank‐order correlation analysis was conducted to examine the associations between changes in developmental domains and play skills. Significant negative correlations were found between improvements in gross motor development and the manipulation (*r* = −0.776, *p* = 0.023) and attention (*r* = −0.784, *p* = 0.021) domains of play. These findings indicate that greater gains in gross motor development were associated with lower scores in manipulation and attention components of play. No other significant correlations were observed between developmental domains and play skill measures.

Overall, these findings demonstrate that online occupational therapy interventions implemented through a telerehabilitation‐based model significantly enhanced both developmental and play‐related skills in children with CVI. The strong correlations between developmental progress and play skill enhancements further support the effectiveness of the intervention in promoting functional outcomes in this population.

## 4. Discussion

The findings of this study indicate that online occupational therapy interventions delivered via telerehabilitation significantly improve developmental and play skills in children with CVI. This study aligns with previous research emphasizing the importance of structured interventions and environmental modifications in facilitating developmental progress in children with visual impairments [4]. The improvements observed in language–cognitive, fine motor, and gross motor skills following the intervention highlight the effectiveness of play‐based approaches within the PEO model [10].

One of the most significant outcomes of this study was the enhancement in play behaviors, as measured by the RKPPS. Play is a crucial aspect of child development, and children with CVI often exhibit delays in object manipulation, symbolic play, and social participation [6]. The results showed that symbolic play and participation increased significantly, suggesting that structured online interventions can bridge the gap in social and imaginative play behaviors. Similar findings have been reported in previous studies emphasizing the role of structured play‐based interventions in promoting social interaction and independent play in children with visual impairments [5, 7].

The strong correlations observed between developmental skills and play abilities further support the idea that cognitive and motor improvements directly contribute to more effective play interactions. Specifically, the relationship between language–cognitive development and symbolic play suggests that higher cognitive function enables more advanced play scenarios, supporting previous findings in developmental psychology and occupational therapy [10, 11]. Additionally, the association between fine motor skills and material management highlights the importance of dexterity in engaging with play objects, which is consistent with previous research on motor function and play engagement in children with disabilities [9].

The telerehabilitation model used in this study has notable clinical implications, especially for children with limited access to in‐person therapy. Telerehabilitation provides a cost‐effective and accessible alternative for delivering high‐quality occupational therapy interventions to children with neurological and visual impairments [8]. The findings support the growing acceptance of digital health solutions in pediatric occupational therapy, emphasizing the potential of remote interventions to overcome geographical and logistical barriers.

However, this study has some limitations. First, the sample size was relatively small, limiting the generalizability of findings. Future studies with larger cohorts are needed to validate these results. Second, while the study duration was 8 weeks, longer‐term follow‐ups are necessary to assess the sustainability of improvements in developmental and play skills. Additionally, the study relied on parent‐reported compliance with at‐home interventions, which may introduce bias in measuring the effectiveness of the therapy. Future research should incorporate objective monitoring tools to track engagement and progress in remote therapy settings. The absence of standardized neuropsychological testing represents a limitation; however, functional developmental assessments and clinical records were used to characterize participants′ cognitive profiles. The absence of a control group limits causal inference; however, the pretest–posttest design allowed for within‐subject comparison of intervention effects.

In conclusion, this study provides strong evidence that online occupational therapy interventions, structured within the PEO model, can significantly enhance developmental and play‐related skills in children with CVI. The results emphasize the importance of structured, individualized therapy approaches and highlight telerehabilitation as a viable model for delivering effective occupational therapy interventions. Future research should explore long‐term outcomes, optimize intervention protocols, and expand the use of digital therapy solutions for pediatric populations with visual and neurological impairments.

## 5. Conclusion

This study demonstrated that online occupational therapy interventions, structured within the PEO model, can significantly enhance developmental and play skills in children with CVI. The results showed notable improvements in language–cognitive, fine motor, and gross motor skills, alongside significant gains in symbolic play, material management, and play participation. These findings highlight the effectiveness of play‐based interventions delivered via telerehabilitation, reinforcing the potential of remote occupational therapy as a viable alternative for children with limited access to in‐person services.

The strong correlations between developmental progress and play engagement further emphasize the interdependent nature of these domains. Enhancing cognitive and motor abilities directly contributes to greater participation in structured and unstructured play activities, underlining the importance of comprehensive therapy approaches that integrate both developmental and occupational components.

Despite these promising findings, future research should focus on larger sample sizes and long‐term follow‐ups to validate the sustainability of observed improvements. Additionally, the integration of objective tracking tools and parental training modules in telerehabilitation‐based interventions may further optimize therapy outcomes.

In conclusion, this study supports the growing adoption of telerehabilitation in pediatric occupational therapy, demonstrating that targeted online interventions can effectively foster developmental and play‐related skills in children with CVI. The findings contribute to the evolving landscape of digital health solutions, advocating for innovative and accessible approaches in occupational therapy practice.

## Funding

No funding was received for this manuscript.

## Disclosure

All authors have read and approved the final version of the manuscript and consent to its publication.

## Conflicts of Interest

The authors declare no conflicts of interest.

## Data Availability

The datasets used and/or analyzed during the current study are available from the corresponding author on reasonable request.
